# Music Interventions and Child Development: A Critical Review and Further Directions

**DOI:** 10.3389/fpsyg.2017.01694

**Published:** 2017-09-29

**Authors:** Elisabeth Dumont, Elena V. Syurina, Frans J. M. Feron, Susan van Hooren

**Affiliations:** ^1^Music in Education, Zuyd University of Applied Science, Maastricht, Netherlands; ^2^Health, Ethics and Society, Maastricht University, Maastricht, Netherlands; ^3^Faculty of Science, Athena Institute, Vrije Universiteit, Amsterdam, Netherlands; ^4^Social Medicine, Maastricht University, Maastricht, Netherlands; ^5^Healthcare, Zuyd University of Applied Science, Maastricht, Netherlands

**Keywords:** music education, child developmental outcomes, child development, review, music

## Abstract

Research on the impact of music interventions has indicated positive effects on a variety of skills. These findings suggest musical interventions may have further potential to support educational processes and development of children. This paper reviews the latest evidence on the effect of musical interventions on the development of primary school-aged children. Four electronic databases were searched from January 2010 through June 2016 using *music, music instruction, music education, music lesson, music training, development, child, student*, and *pupil* as key words for the search. *Two* reviewers independently evaluated the studies to determine whether they met the stated inclusion criteria. Studies were compared on study setup, methodological quality, intervention components, outcome variables, and efficacy. A review of these selected studies (*n* = 46) suggestive beneficial effects of music intervention on development of children, although clear conclusions cannot be drawn. Possible influencing factors that might contribute to the outcome of intervention are reviewed and recommendations for further research are made.

## Introduction

Music interventions are often said to have an influence on motor, language, social, cognitive, and academic abilities (Ho et al., 2003; Costa-Giomi, 2004; Schellenberg, 2004; Forgeard et al., 2008; Standley, 2008; Jentschke and Koelsch, 2009; Southgate and Roscigno, 2009; Yazejian and Peisner-Feinberg, 2009; Strait et al., 2010). Music may play an important role in meeting a child's educational needs as it provides a means of self-expression, giving the child an outlet for feelings and emotions. Music, aside from being a source of enjoyment, is also a means of communication with others (Suthers and Niland, 2007). Music may expose the child to challenges and multi-sensory experiences which enhance learning abilities and encourage cognitive development. In particular, music can also engage cognitive functions, such as planning, working memory, inhibition, and flexibility. These functions are known as executive functions (EF). Although there is no consensus on conceptualization, there is agreement on the complexity and the importance of EF for learning and development (Gioia et al., 2000). Music education may be a promising tool in improving EF as it activates multiple cortical and subcortical brain areas, including the prefrontal cortex, which is linked to EF (Särkämö et al., 2014).

Musical interventions may become an appealing approach for schools that are increasingly facing a challenge of supporting education processes and development of children with varied degrees of learning and behavioral difficulties. However, before an extended use can be introduced into practice, we need to have a clearer, more systematic understanding of the known effects musical interventions have.

The current study builds on the results of previous reviews of literature examining the impact of music training and education including, among others, those of Jaschke et al. (2013), Cogo-Moreira et al. (2012), Besson et al. (2011), Maloy and Peterson (2014), and Miendlarzewska and Trost (2014). Jaschke et al. (2013) found mixed evidence of far transfer effects between music education and other cognitive skills. Cogo-Moreira et al. (2012) aimed to review RCTs to investigate the effectiveness of music education on reading skills in children and adolescents with dyslexia but were unable to find such studies. In a meta-analysis, Maloy and Peterson (2014) concluded that there was a minimal effect of music as an intervention to increase task performance in children and adolescents with ADHD. Miendlarzewska and Trost (2014) found that musical training in childhood has a positive impact on many cognitive functions and is associated with neuroplastic changes in brain structure and function. The transfer of training from music to speech was evaluated by Besson et al. (2011), who pointed to positive transfer of training effects from musical expertise to speech processing. When interpreting the results, it is important to take into consideration that these reviews in general yielded mixed results and were limited in their focus: specific skills (Cogo-Moreira et al., 2012), a specific developmental domain (Miendlarzewska and Trost, 2014), specific designs and age groups (Cogo-Moreira et al., 2012; Jaschke et al., 2013), or a specific target group (Maloy and Peterson, 2014).

Bearing these in mind, the purpose of this article is to provide a comprehensive summary of the existing research in the field by collecting and analyzing the latest evidence on the effect of music interventions across different domains of development of the primary school-aged children. It aims to report on the effectiveness of a broad range of music interventions, describe relevant contextual factors, to evaluate the general level and quality of evidence in the field and to provide implications for future research.

## Methods

Due to a broad scope of this study, we decided to do a systematic search and a “critical review,” which aims to “extensively research the literature and critically evaluate its quality” (Grant and Booth, 2009). Several steps were taken in order to ensure high scientific quality of the work.

### Search procedure

**Figure 1:**
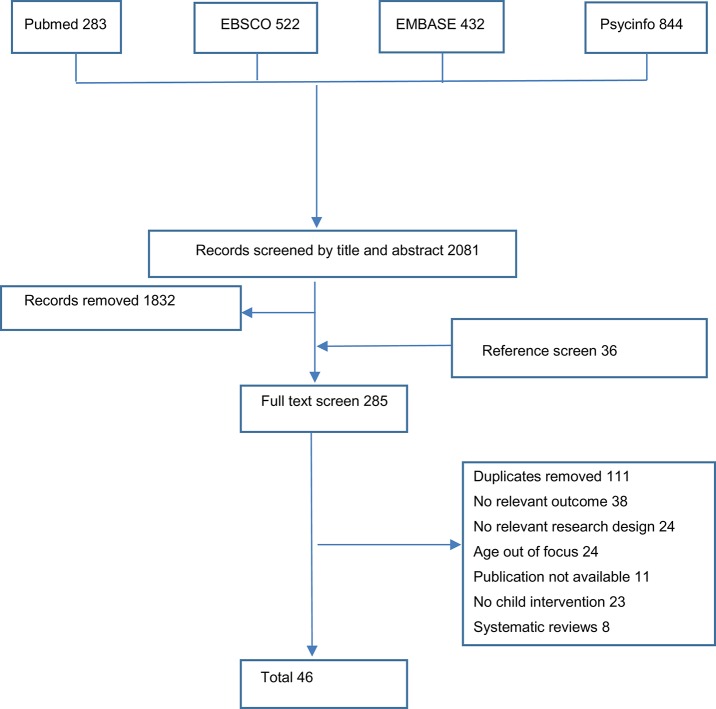
Flow diagram of article identification and inclusion.

The search for relevant articles was conducted via three routes. First, PubMed, EMBASE (Ovid), PsycInfo, and EBESCO databases were systematically searched. The search covered 6 years (January 2010 to June 2016) and the following search terms were included: music, music education, music instruction, music lesson, music training, development, child^*^, student, pupil. The key-words were combined in various ways using Boolean terms AND and OR. Second, reference lists of the identified relevant systematic reviews and key articles (referenced by more than 1 paper) were examined in order to identify additional studies. The last route included a manual search of the tables of contents of relevant journals: International Journal of Music Education and British Journal of Music Education. A flowchart describing these processes is reported in Figure 1.

### Inclusion and exclusion criteria

Identified studies were considered eligible for inclusion if they met the following a priori defined criteria. The studies had to (a) involve training, teaching, or providing intervention using music; (b) utilize outcome measures targeting child's development; (c) focus on the (pre)school-aged children up to 13 years without physical disabilities; (d) be published in a peer-reviewed journal between January 2010 and June 2016; (e) be written in English. We excluded studies that (a) examined use of music psychotherapy interventions; (b) focused only on imaging techniques, (c) had musical outcomes only; (d) were not based on empirical data: qualitative reviews, commentaries, case studies or studies without an accurate methodological description.

### Screening and study selection

Upon removal of the duplicates, the literature search yielded 1,092 results. All identified studies were subjected to multilevel screening, executed independently by two co-authors (ED and EVS). First, the titles and abstracts of identified studies were screened. At this stage, the titles and abstracts that did not meet at least one inclusion criteria (non-English language, commentaries) were omitted. Based on this first screening, 126 potentially relevant articles were obtained as full texts. Next, these articles were further reviewed by ED and EVS independently to determine whether or not they met the stated inclusion criteria. All exclusion decisions were documented. Each reviewer made a selection list, which were then compared. In cases of disagreement, the articles in question were discussed by all co-authors and a consensus decision was made. Our final selection included 46 articles.

### Data analysis

Studies that met at least one inclusion criteria, but did not meet any of the exclusion criteria, have been reported according to a list of five variables in order to extract data in a comparable way. The methodological quality of the studies was assessed using the guidelines of the Dutch Institute for Health Care Improvement (CBO). The following elements were evaluated: randomization, allocation concealment, baseline comparability, blinding of participants or providers, blinding of outcome assessors, reporting of attrition rate, the use of intent-to-treat analyses and the use of validated tools. The level of evidence of each study was determined according to the guidelines of Melnyk and Fineout-Overholt (2005).

## Results

The main results of selected studies are reported in Table 1. All studies involved participants of 4–13 years-old, but some were not limited to this range: two studies chose a broad age range of 6–25 years [35] and 6–59 years [32]. Sample sizes varied between 10 [1] and 352 [35]. In general, studies employed both genders. One study [40] and one sub-experiment of a study [42] included males only. Although the type of design was not explicitly mentioned in all studies, most studies implemented a(n) (quasi-) experimental, longitudinal, or correlational design. Only three of the 46 studies used a randomized control trial (RCT) [21] [29] [17].

**Table 1:** Intervention details for included studies.

**Number**	**Study (Country)**	**Number, age, and sex of participants**	**Research design**	**Level of evidence**	**Domain**	**Dependent variable(s)**	**Results**	**Effect size reported**
[1]	Brodsky and Sulkin, 2011 (Israel)	EXP 1 *N* = 18 88% girls Mean age 7 years	Two-group pretest– posttest intervention	III	Motor skill development/cognitive development	Non-music motor and cognitive abilities	+	
		EXP 2 *N* = 10 40% girls Age 8–8.5 years						EXP 2 η^2^*_*p*_* ranged from 0.4081 to 0.6512
		EXP 3 *N* = 51 60% girls Age 8 years						EXP 3 η^2^*_*p*_* ranged from 0.0767 to 0.5354
[2]	Janzen et al., 2014 (Australia)	*N* = 57 32 females Age 10–14 years	Quasi-experimental	III	Motor skill development	Timing skills involved in the control of discrete and continuous movements	+	No information
[3]	Kirschner and Tomasello, 2010 (Germany)	*N* = 96 48 females Mean age 4.6 years	Between participants design	III	Social development	Voluntary helping and spontaneous cooperative problem solving	+	No information
		Random assignment to groups						
[4]	Ritblatt et al., 2013 (USA)	*N* = 102 49 females Age 36–60 months	Quasi-experimental	III	Social development	Social skills and school readiness	±	No information
[5]	Schellenberg et al., 2015 (Canada)	*N* = 84 49 females Mean age 8 years and 8 months	Natural experiment	III	Social development	Prosocial skills	±	η^2^*_*p*_* ranged from 0.058 to 0.546
[6]	Rabinowitch et al., 2013 (UK)	*N* = 52 28 girls Age 8–11 years	Experimental	III	Social development	Emotional empathy	±	No information
		Random assignment to groups						
[7]	Schellenberg and Mankarious, 2012 (Canada)	*N* = 60 28 boys Age 7–8 years	Quasi-experimental	III	Social development	Emotion comprehension	–	η^2^*_*p*_* ranged from 0.12 to 0.30 η^2^ ranged from 0.12 to 0.19
[8]	Rickard et al., 2013 (Australia)	*N* = 195 Mean age younger cohort 5.92–6.06 years Mean age older cohort 8.65–8.69 years	Experimental	III	Social development	Self-esteem and social skills	±	η^2^*_*p*_* ranged from 0.014 to 0.083
[9]	Degé et al., 2014 (Germany)	*N* = 92 47 boys Age 12–14 years	Correlational	III	Social development	Global academic self-concept	+	No information
[10]	Bhide et al., 2013 (UK)	*N* = 19 11 males Mean age 6.7–6.9	Intervention study	III	Language	Auditory, phonological and literacy skills	–	Effect sizes (*d* or *r*) ranged from 0.44 to 2.27 (based on pre- and post-test measures of the musical intervention group)
[11]	Degé and Schwarzer, 2011 (Germany)	*N* = 41 Mean age 5.6 years	Pretest-posttest design	III	Language/cognitive development	Intelligence and phonological awareness	–	No information
		Random assignment to groups						
[12]	Escalda et al., 2011 (Brazil)	*N* = 56 33 females Age 5.0–5.11 years	Descriptive-comparative	III	Language	Auditory processing abilities and phonological awareness	+	OR ranged from 0.33 to 30.39
[13]	Herrera et al., 2011 (Spain)	*N* = 97 44 females Mean age 4.5 years	Two year pretest/posttest study	III	Language/cognitive development	Phonological awareness, verbal short term memory, and naming speed	–	η^2^ ranged from 0.07 to 0.67
		Random assignment to groups						
[14]	Fonseca-Mora et al., 2015 (Spain)	*N* = 63 34 females Mean age 7.6 years	Pre-post comparison design	III	Language/cognitive development	Early reading skills, working memory, and decoding skills	–	η^2^*_*p*_* ranged from 0.034 to 0.354
[15]	Moritz et al., 2013 (USA)	EXP 1 *N* = 30 13 girls Mean age 5.6 years	Exploratory	III	Language	Phonological awareness and acquisition of reading skills	+	*r* ranged from –0.06 to –0.40
		EXP 2 *N* = 16 6 girls Mean age 8.1 years						
[16]	Moreno et al., 2011b (Canada)	*N* = 60 26 boys Age 4–6 years	Pretest/training/posttest design	III	Language	Preliteracy skills	+	No information
		Pseudo-random assignment to groups						
[17]	Flaugnacco et al., 2015 (Italy)	*N* = 48 8–11 years old	RCT	II	Language	Linguistic, musical, reading and general cognitive abilities	±	Effect sizes ranged from 0.3 to 0.4
		Pseudo-random assignment to groups						OR ranged from 1.9 to 3.7
[18]	Habib et al., 2016 (France)	EXP 1 *N* = 34 Age 8.2–11.7 years Controls 30 months younger on average	Quasi-experimental	III	Language	Different aspects of auditory and speech perception	+	
		EXP 2 *N* = 12 Age 7–12 years						EXP 2 *d* ranged van 0.52 to 3.09
[19]	Corrigall and Trainor, 2011 (Canada)	*N* = 46 35 girls Age 6–9 years	Correlational	III	Language	Reading skills	±	No information
[20]	Bonacina et al., 2015 (Italy)	*N* = 28 8 females Mean age 12.07 years	Test-training-retest experimental design	III	Language	Reading accuracy and reading speed and rhythmic perception skills	±	η^2^ ranged from 0.145 to 0.660
		Random assignment to groups						
[21]	Cogo-Moreira et al., 2013 (Brazil)	*N* = 235 from 10 schools 38.3% females Mean age 9.15 years Random assignment to groups	RCT	II	Language/academic performance	Academic achievement in Portuguese and math and phonological awareness and reading	±	No information
[22]	Slater et al., 2014 (USA)	*N* = 42 26 females Age 6–9 years Pseudo-random assignment to groups	Longitudinal	III	Language	English reading ability	+	η^2^*_*p*_* ranges from 0.118 to 0.153
[23]	Rautenberg, 2013 (Germany)	*N* = 159 80 girls Mean age 7.7–7.11 years	Experimental	III	Language	Decoding skills in word reading and musical ability	±	No information
		Random assignment to groups						
[24]	Schellenberg, 2011 (Canada)	*N* = 106 52 girls Age 9–12 years	Quasi-experimental	III	Cognitive development	IQ and executive functions	±	No information
[25]	Moreno et al., 2011a (Canada)	*N* = 64 26 boys Age 4–6 years	Longitudinal	III	Cognitive development	Verbal and spatial intelligence and executive functions	+	η^2^*_*p*_* ranged from 0.09 to 0.33
		Pseudo-random assignment to groups						
[26]	Bugos and Jacobs, 2012 (USA)	*N* = 28 Mean age 11.20–11.23 years	Quasi-experimental	III	Cognitive development	Music reading, processing speed, vocabulary performance, verbal fluency, and arithmetic computation	±	*d* = 0.33 (arithmetic subtest)
[27]	Roden et al., 2014 (Germany)	*N* = 345 190 females Mean age 7.87 years	Quasi-experimental	III	Cognitive development	Auditory cognition, visual attention and processing speed	±	*d* ranged from 0.24 to 1.25
[28]	Kaviani et al., 2014 (Iran)	*N* = 60 32 females Age 5–6 years	Experimental	III	Cognitive development	IQ	+	Effect sizes (*SMD*) ranged from 0.12 to 0.75
		Random assignment to groups						
[29]	Mehr et al., 2013 (USA)	EXP 1 *N* = 29 13 females Mean age 4.64–4.86 years	RCT	III	Cognitive development	Spatial-navigational reasoning, visual form analysis, numerical discrimination, and receptive vocabulary	–	EXP 1 *d* ranged from 0.65 to 0.72 (navigation test and visual form analysis test)
		EXP 2 *N* = 45 21 females Random assignment to groups						Combined analyses of EXP 1 and 2 revealed no significant effects
[30]	Rickard et al., 2010 (Australia)	Mean age 8.62–8.79 years	Longitudinal/quasi-experimental	III	Cognitive development	Verbal and visual learning and memory	±	η^2^*_*p*_* ranged from 0.029 to 0.101
[31]	Degé et al., 2011 (Germany)	*N* = 34 22 girls Mean age 10.10 years	Longitudinal	III	Cognitive development	Visual short term memory and auditory short term memory for environmental sounds	+	η^2^ ranged from 0.12 to 0.33
[32]	Martens et al., 2011 (USA)	EXP 1 *N* = 38 22 females Age 6–59 years	Experimental	III	Cognitive development	Verbal short term and long term memory	±	EXP 1 η^2^*_*p*_* = 0.19
		EXP 2 *N* = 38 25 females Age 7–50 years						
[33]	Roden et al., 2012 (Germany)	*N* = 73 Mean age 7.73 years	Quasi-experimental	III	Cognitive development	Verbal and visual memory	±	η^2^*_*p*_* ranged from 0.07 to 0.34 *d* ranged from 0.49 to 1.35
[34]	Roden et al., 2014 (Germany)	*N* = 50 Age 7–10 years	Quasi-experimental longitudinal design	III	Cognitive development	Performance on visual sketchpad measures, phonological loop measures, and central executive measures	±	η^2^*_*p*_* ranged from 0.07 to 0.30 *d* ranged from 0.43 to 1.33
[35]	Bergman Nutley et al., 2014 (Sweden)	*N* = 352 168 females Age 6–25 years	Longitudinal	III	Cognitive development/academic performance	Working memory, speed processing, non-verbal reasoning and academic tests	±	No information
[36]	Portowitz et al., 2014 (USA/Israel)	*N* = 84 9–10 year	Mixed methods	III	Cognitive development	Working memory, self-regulation and cognitive flexibility	+	η^2^ = 0.11
[37]	Khalil et al., 2013 (USA)	*N* = 102 56% females Age 7–12 years	Correlational	III	Cognitive development	Attention behavior	+	No information
[38]	Zuk et al., 2014 (USA)	*N* = 27 11 males Mean age 10.9 years	Cross-sectional	III	Cognitive development	Executive function measures	+	No information
[39]	Janus et al., 2016 (Canada)	*N* = 72 4–6 years old Pseudo-random assignment to groups	Longitudinal intervention design	III	Cognitive development	Cognitive performance	–	η^2^*_*p*_* ranges from 0.07 to 0.80
[40]	Pelham et al., 2011 (USA)	EXP 1 *N* = 67 All boys Mean age 9.8 years	Quasi-experimental	III	Academic performance	Rules violations, teacher prompts, seatwork completion, and on task behavior	±	EXP 1 *d* ranged from −1.30 to 1.64
		EXP 2 *N* = 86 All boys Mean age 9.47 years						EXP 2 *d* ranged from −0.84 to 0.52
[41]	Courey et al., 2012 (USA)	*N* = 67 Age 8.5–10.11 years	Quasi—experimental comparison group pretest/posttest design	III	Academic performance	Music notation, fraction symbols and fraction size	±	η^2^*_*p*_* ranged from 0.10 to 0.12 *d* ranged from 0.44 to 1.46
[42]	Rickard et al., 2012 (Australia)	EXP 1 *N* = 111 All male Mean age 12.67 years	Experimental	III	Social development/cognitive development/academic performance	Verbal memory, intelligence, self-esteem, attitudes to school and motivation & engagement with classes, aggression, social skills, depression and academic measures	–	EXP 1 η^2^ ranged from 0.05 to 0.273
		EXP 2 *N* = 106 58 females Mean age 131.07 months Random assignment to groups						EXP 2 η^2^*_*p*_* ranged from 0.081 to 0.084
[43]	Yang et al., 2014 (China)	*N* = 250 122 females Mean age onset 78 months	Longitudinal	III	Academic performance	First and second language (L1: Chinese, L2: English) and mathematical skills	±	No information
[44]	Swaminathan and Gopinath, 2013 (India)	*N* = 76 55 females Mean age 98.99–100.55 months	Quasi-experimental	III	Academic performance	Second language verbal and readings skills	+	*d* ranged from 0.52 to 0.63
[45]	François et al., 2013 (France)	*N* = 24 14 boys Mean age 8 years Pseudo-random assignment to groups	Longitudinal	III	Other, non-musical skills	Speech segmentation	+	No information
[46]	Slater et al., 2015 (USA)	*N* = 38 21 females Mean age 97.32–100.05 months Random assignment to groups	Longitudinal	III	Other, non-musical skills	Speech in noise perception	+	η^2^ = 0.110 *d* ranged from 0.3013 to 1.140

The reviewed articles have spawned a broad range of approaches to and considerable heterogeneity in music interventions. In general, music interventions consisted either of structured musical instruction/activities, i.e., use of instruments, singing, moving, listening, improvising, music notation, rhythm training, composing music, instrumental classes, or private instrumental training. Only in several studies, the music intervention was especially designed for the acquisition of specific non-musical skills [10] [11]. Length of the intervention varied across studies, ranging from seven and a half minutes [31] to 11 semesters [43]. Music interventions were mostly provided two or three times per week. In three studies, interventions were delivered on a daily basis [15] [16] [25]. All but two studies [32] [40] used live music as opposed to recorded music. Four studies used a specific pedagogical approach for music instruction: the Orff method, which refers to a way of teaching children about music that engages their mind and body through a mixture of singing, dancing, acting, and the use of percussion instruments or the Kodaly method, in which children are first introduced to concepts of music through experiences such as singing, listening or movement. Only after the child becomes familiar with the concept of music do they learn how to compose it [8] [15] [28] [45]. Interventions were either performed in (small) groups or individual (in case of instrumental training). The authors conducted studies either in the school/classroom environment, where music interventions would be regularly conducted; or used locations outside school i.e., music schools or specific center for music teaching. Information about the person who delivered the music intervention was mentioned in 30 studies. In most studies a professionally trained music teacher was employed. In two other studies, the intervention was delivered by parents/teachers [4] who received training or by trained research assistants [11]. In four studies, the intervention was either computer-based [16] [25] or delivered via CD/radio [32] [40].

The reviewed articles used varied outcome measures affected by music interventions. Outcome measures can be grouped in the following categories: motor skill development, social and emotional development, language, cognitive development, academic performance and other, non-musical, related skills.

### Motor skill development

We identified no studies that focused particularly on the association between music training and gross motor skill development. Two studies explored, among others, the beneficial impact of music activity on specific motor skills. Using a non-randomized design, Brodsky and Sulkin (2011) [1] (which presented results of three experiments) focused on hand-clapping songs. In the first experiment, the association of performance quality of handclapping songs with academic achievement was evaluated among a class of 18 children (mean age 7 years). Two handclapping songs were taught by rote via live demonstration by the second author during a 3-week period and both performance quality and achievement of all 18 children were assessed. Results indicated that children who were more skillful in performing handclapping songs, were also more efficient learners. In a second experiment, the authors measured bimanual rhythmic patting and aural diction in 10 children aged 8–8.5 years, five children who self-reported engagement in handclapping songs activity, and five children from the same classroom who self-reported not to engage in handclapping songs were recruited. Self-reports were confirmed by the second author through observations. The authors found that children who spontaneously engaged in hand clapping songs had an advantage in aural diction and accuracy performance of eye-hand motor sequences. The third experiment took place over 8 weeks. Twenty-four children received classroom handclapping intervention (HCST) while another 27 received the music appreciation guided listening curriculum (MAGL). Children who received HCST were more effective in developing bimanual coupling, writing proficiencies and handwriting compared to children who received MAGL (Brodsky and Sulkin, 2011 [1]). Janzen et al. (2014) [2] investigated whether formal music training enhances precision in discrete and continuous movements. The study included 32 children enrolled in music classes who had at least 2 h of weekly musical activities. Twenty-five children who were not involved in any musical activity were also included. All were 10–14 years-old. Results showed that musically trained children had a significantly more accurate performance in the discrete movement task compared to controls. Findings suggest performance was positively associated with the number of years of formal music training. Musically trained children also tended to be more precise in the continuous movement task (Janzen et al., 2014).

Although reporting positive results, a limitation of above-mentioned, quasi-experimental studies was the lack of randomization. In a sub-experiment of one study only (sub experiment 3) [1], participants were matched socioeconomically and an active control group was included. In the second study of another sub-experiment, performance of the music group was compared to control groups who were not involved in music training [2] or who did not receive any additional activity (sub experiment 2) [1]. Therefore, caution should be used when making inferences about the observed effects of the music interventions on specific motor skills.

### Social and emotional development

#### Social skills

Four studies reported mixed evidence of the influence of music interventions on social skills. Using a quasi-experimental design, Ritblatt et al. (2013) [4] found that 55 children, aged 3–5, who received a music intervention program focused on socioemotional skills, demonstrated a positive change in these skills compared to a wait-list control group (*n* = 47) who did not receive the music intervention. These changes occurred over the course of a 8-month period. It's important to note that these effects were reported by teachers and not parents. Schellenberg et al. (2015) [5] investigated whether social benefits were accrued from an existing group music training program that was designed with music pedagogy as its focus in 84 8–9 year-old children. Results showed that children in the music group (*n* = 38), who attended schools that incorporated an enhanced group music program into the curriculum, had larger increases in sympathy and prosocial behavior compared to children in the control group (*n* = 46), who attended schools without the enhanced music program, but this effect was limited to children who had poor prosocial skills before the lessons began. Evidence from a between-participants study of the effects of joint music making in 48 pairs of 4 year-old children [3] who were randomly assigned either to the music condition (i.e., episode of interactive play with joint music making) or the non-music condition (i.e., episode of interactive play without music), demonstrated an increase in willingness to help one another and to cooperate on a problem-solving task in children in the music condition compared to non-music condition (Kirschner and Tomasello, 2010) [3]. However, in an experimental study, Rickard et al. (2013) [8] assigned 195 5–8 year-old children to either a music education (*n* = 122) or a control group (*n* = 73) based on the school they were attending. Children receiving a music education received age-specific, specialized music programs on top of the preexisting, general school music program, while children in the control group did not receive these specialized music programs but continued with their regular school music program. The authors found no benefits of the specialized music program on children's social skills compared to children in the control group.

In sum, three studies [3] [4] [5] reported partially positive results, whereas one study [8] reported no effects. One study reporting a beneficial impact of music [3] is of high quality$ i.c. incorporating random assignment to conditions, blinding the outcome assessors and incorporating an active, matched control program without music. The intervention lasted, however, for 20 min. The partially positive findings of Ritblatt et al. (2013) [4] and Schellenberg et al. (2015) [5] should be interpreted with caution due to the design used [5], the lack of randomization, the fact the sample may not be representative of the target population (i.c. higher SES and higher educational level) [4] and teacher/parent expectations which may have influenced the results [4]. In the experimental study [8] of Rickard et al. (2012) [8], reporting no effects of a specialized music program on top of the general school music program, randomization was absent. However, the relatively large sample size, the duration of the study and the inclusion of an active control group are strengths of this study.

Results of above mentioned studies are mixed and demonstrate the need for further research.

#### Emotional development

Two studies addressed the influence of music on emotional development and reported mixed results. A study of Schellenberg and Mankarious (2012) [7] assessed 60 children, ranging from 7 to 8 years-old, on a test of emotion comprehension (TEC). The musically trained group included 30 children who had at least 8 months of formal music lessons taken outside the school, whereas the untrained group consisted of 30 children who had no music training outside the school. Musically trained children demonstrated significantly higher TEC scores than the ones without music training. The effect remained even after accounting for demographic variables. However, the link appeared to be a consequence of high level cognitive functioning of the musically trained group. No group differences were present when IQ scores were accounted for. Using an experimental study, Rabinowitch et al. (2013) [6] tracked 52 children aged 8–11 after they were randomly assigned to either a musical group interaction program (*n* = 23), a games group (*n* = 8), receiving a similar program without the use of music or a control group (*n* = 21), not receiving any special activity. Children in the music group showed an increase in empathy scores on two out of three measures compared to children in the games group and children in the control group.

While both studies reported positive results, the findings of the study of Schellenberg and Mankarious (2012) [7] turned out to be a related to the level of cognitive functioning of participants in the music group. The experimental study of Rabinowitch et al. (2013) [6] permits, at least to some extent, for causal inference. The authors used randomization to allocate participants to conditions, thereby reducing the risk of bias from confounding. The small sample size and the fact that the active and the passive control group were merged into one control group before comparison with the music group, should, however, be taken into consideration. Based on findings from both studies, no definitive conclusions can be drawn yet and more research is needed in this area to achieve conclusive results.

#### Academic self-concept, psychosocial wellbeing, and self-esteem

Three studies reported mixed effects of music on academic self-concept, which refers to the cognitive representation and appraisal of one's own abilities in academic performance (Degé et al., 2014), psychosocial wellbeing and self-esteem, which describes one's overall sense of self-worth. In a correlational study, Degé et al. (2014) [9] revealed that duration of music lessons was positively associated with academic self-concept in 92 12–14 year-old children, even after controlling for demographic variables and IQ. In a 3 year experimental study, Rickard et al. (2013) [8] showed that increase in school-based music lessons prevented a decline in global self-esteem measures experienced by the control group in both the younger and older cohorts across the first year of the study. However, effect sizes were generally modest in the second year. In another study, Rickard et al. (2012) [42] investigated the effect of increasing existing music education (study 1) and the effect of introducing a novel high-quality music education program (study two) on various psychosocial measures in 111 10–13 year-old children (all males). One hundred eleven 10–13 year-old children in study one were pseudo-randomly assigned to additional music classes (*n* = 47), art classes (*n* = 27), or drama classes (*n* = 37). One hundred six children in study two (mean age 131.07 months) were randomly allocated to a music group (*n* = 38), a drama group (*n* = 37) or control group, receiving no program (*n* = 31). No significant effects were found.

Degé et al. (2014) [9], using a correlational design, was the only one reporting positive results. However, these results do not allow for any conclusions to be drawn about causality. Two experimental studies of Rickard et al. (2012) [8] [42] found modest effects and no effects, respectively music interventions on top of the preexisting school music education. The (relatively) large sample sizes [8] [42] and the duration of one the studies [8] can be considered as strengths. Both studies, did not, however, randomize participants to the intervention or control groups. In one of the two experimental studies [42], active control groups were included, who continued their regular school music program. The other study [8] included both passive and active control groups, which better allowed for comparison of the increased music education.

In summary, although one study reported positive correlations, two studies suggest little or no beneficial effect. Further research is needed to clarify whether music can positively impact self-concept, self-esteem, and psychosocial wellbeing.

### Language

Studies that link music intervention to language acquisition can be clustered into two groups: (1) focus on phonological awareness and auditory processing and (2) reading.

#### Phonological awareness and auditory processing

Several studies assessed the influence of music on auditory and phonological skills with mixed findings. Some suggest that musical activities have a beneficial effect on these skills. Using a descriptive-comparative design, Escalda et al. (2011) [12] examined the relationship between musical experience, auditory processing abilities and phonological awareness skills of 56 five year-old children. Results showed that 26 children, with musical experience, performed significantly better on auditory processing and phonological awareness than 30 children without musical experience. In an exploratory study, Moritz et al. (2013) [15] investigated whether musical activity could enhance the acquisition of reading skill, potentially before formal reading instructions began in 30 children (mean age 5.6 years). Children in the music group (*n* = 15) received daily 45 min music lessons whereas children in the control group (*n* = 15) received weekly 35-min music lessons. Correlational results showed that rhythm ability was related to phonological segmentation skills at the beginning of kindergarten and that end-of-year phonological awareness skills of children who received daily music lessons were better than skills of children in the control group who received music lessons once a week. Using a pragmatic RCT, Cogo-Moreira et al. (2013) [21] included 235 participants with reading problems, aged 8–10 years, in 10 schools, to compare the effectiveness of music education for the improvement of among other, reading skills. Five schools were randomly chosen to incorporate music classes (*n* = 114) and five schools, who were not encouraged to offer musical activities, served as controls (*n* = 121). There was no improvement in phonological awareness when comparing the two groups. Flaugnacco et al. (2015) [17], also using an RCT, pseudo-randomly assigned 8–11 year-old dyslexic children to a music group (*n* = 24) or a painting group (*n* = 24). Both groups also received conventional rehabilitation program. After 7 months of training, the music group outperformed the painting group in tasks assessing rhythmic abilities and phonological awareness. Using a pretest/training/posttest design, Moreno et al. (2011b) [16] focused on the effects of an intensive computerized training in music or visual arts on pre-literacy skills in 60 4–6 year-old children, who were pseudo-randomly assigned to the music or visual arts condition. They reported comparable improvements in both groups in rhyme awareness and in ability to map unfamiliar symbols to known words. However, when the two groups were statistically equated at pretest, the magnitude of improvement was found to be larger in the music group. Herrera et al. (2011) [13] on the other hand, used a 2 year pretest-posttest study in which 97 children (mean age 4.5 years) at two preschools were allocated following stratified randomization procedures into a group that received phonological training with music (*n* = 32), a group that received phonological training with no music (*n* = 34) and a control group who did not receive any specialized training (*n* = 31). Phonological training was effective regardless of whether it included music and whether the children were foreign Spanish speakers or native speakers. Both experimental treatment groups outperformed the control group in the posttests on phonological awareness tasks and speed in naming objects. However, the phonological training with music group outperformed the phonological training without music group on phonological awareness of ending sounds. In general, the foreign Spanish speakers were significantly slower in the naming task than their Spanish counterparts, those who had participated in the training with musical activities outperformed their peers in the control group by the end of the treatment. Bhide et al. (2013) [10] compared the effects of a musical intervention for poor readers (*n* = 10) with a software intervention of known beneficial effects based on rhyme training and phoneme-grapheme learning (*n* = 9) in 6–7 year-old children, all of them identified by their class teachers as struggling readers. The authors found that both interventions were equally effective for literacy acquisition and phonological skills. Habib et al. (2016) [18] examined the effectiveness of a specially-designed Cognitivo-musical training (CMT) in two studies. In study one, 12 children with a diagnosis of severe dyslexia (mean age 10.7 years) received daily 6 h of CMT on 3 consecutive days while 22 reading-age matched normal-reading children (30 months younger on average) served as controls, receiving no CMT. The authors found that dyslexic children were impaired in the identification test of categorical perception, but their performance reached the level of control children after 3 days of CMT. Significant improvement in performance of dyslexic children was also noticed in the syllabic lengthening task. In study two, 12 dyslexic children, grouped according to the severity of their problems received CMT training sessions at school. The 3-h weekly sessions were provided over a period of 6 weeks. Results showed a positive influence of the CMT program on categorical perception and the temporal aspects of speech processing. Also, additional improvements in auditory attention, phonological awareness (syllable fusion) were found. Fonseca-Mora et al. (2015) [14], using a pre-post comparison design, tested the efficacy of a phonological training program aimed at improving early reading skills in 7–8 year-old Spanish children learning English as a foreign language in three groups: an experimental group with phonological non-musical intervention (*n* = 22), an experimental group with musical intervention (*n* = 18) and a control group receiving the traditional teaching program (*n* = 23). The results clearly pointed to the beneficial effects of the phonological teaching approach, but the further impact of the music support was not demonstrated. In a longitudinal, experimental study, Degé and Schwarzer (2011) [11] investigated the effect of a music program on phonological awareness in preschoolers. Forty-one children (mean age 5.6 years) were randomly assigned to a music program (*n* = 13), a phonological skills program (*n* = 13), or a sport group (*n* = 14). Results indicated that 26 children who followed either the music program or the phonological program significantly improved in phonological awareness of large phonological units (words) compared to the sport group who received no intervention. All three groups showed similar development in phonological awareness of small phonological units.

Of the two RCTs reviewed, one found beneficial effects of a music intervention on phonological awareness [17] while the other found no effects [21]. Two experimental studies [11] [13], using (stratified) randomization and including two active control groups [11] and an active and passive control group [13], respectively, reported no beneficial effects of music. Of the four studies that used a quasi-experimental design without randomization, two studies, including an active [10] or both an active and passive control group [14], also found no benefits of music interventions One out of these four studies, reporting positive results, used pseudo-random allocation, included an active control group and blinded outcome assessors [16]. Another study, also describing positive results, matched participants, but did include a passive control group only [18]. Positive results were also reported by two correlational studies [12] [15]. However, these results do not allow for any conclusions to be drawn about causality. Although findings suggest music can positively affect phonological awareness and auditory processing in some situations, clear conclusions cannot be drawn.

### Reading

Eight studies addressed the association between music-related activities and a range of reading skills with inconsistent findings. The results of the study of Cogo-Moreira et al. (2013) [21] indicated no improvement in word accuracy, in-text accuracy and non-word accuracy of children in the music intervention schools compared to the children in control ones. In contrast, the RCT results of Flaugnacco et al. (2015) [17] showed better performance of the music group on reading skills in comparison to the control group. Using an experimental design, Bonacina et al. (2015) [20] randomly assigned 11–14 year-old children to a computer-assisted, rhythmic reading training (RRT) (*n* = 14) or a control group (*n* = 14), for which no specific activity addressed to improve reading skills was carried out. Results indicated that RRT had a positive effect on both reading speed and accuracy. The effect of RRT seemed to be specifically on reading skills, as no difference in rhythm perception between the two groups was found. Moritz et al. (2013) [15] found that kindergarteners' rhythm ability was significantly correlated to their phonological awareness and basic word identification skills in second grade. Using a longitudinal design, Slater et al. (2014) [22] compared reading ability of 42 low-income, Spanish-English bilingual children aged 6-to-9, pseudo-randomly assigned to a group music instruction program outside school or a waiting list control group. Twenty-three children in the music group maintained their age-normed performance on the composite reading measure after 1 year, whereas the performance of 19 children in the matched control group deteriorated over the same period of time, consistent with expected declines in this population. Rautenberg (2013) [23], in an experimental study, measured the correlations between musical skills and decoding skills and the effects of musical training on word-level reading abilities. One hundred fifty-nine seven year-old children were randomly allocated to a special music training program (*n* = 33), a visual arts training program (*n* = 41), or no training program for the period of the study (*n* = 85). Results showed the special music training had a significant effect on reading accuracy in word reading. Additionally, positive correlations were found between rhythmical ability and decoding skills. Tonal skills were not correlated with reading skills. In a correlational study of Corrigall and Trainor (2011) [19], it was shown that duration of music training (i.e., the number of years of training on their primary instrument, plus the number of years of training on any additional instruments) was associated with reading comprehension, but not with word decoding among 46 6–9 year-olds. The findings are in contrast to a longitudinal study from Bergman Nutley et al. (2014) [35] which revealed that practicing a musical instrument was not associated with reading comprehension.

Of the eight studies measuring the effects on reading, two studies used an RCT design with pseudo randomization [17] [21] and blinded outcome assessors [17]. Their findings are contradictory; Flaugnacco et al. (2015) [17] found a positive influence of music, whereas the results of Cogo-Moreira et al. (2013) [21] indicated no effect. Results of two studies that used an experimental design with randomization [20] [23] illustrated potential benefits of a music training program. Of these two studies, one included a passive control group, offering no music training program [20] while the other included both a passive and active control group [23], allowing for a more comprehensive comparison. The results of the longitudinal study of Slater et al. (2015) [46] also point to beneficial effects. However, an active control group could not be included. (Partially) positive correlations were shown by two studies [15] [19]. However, correlational studies do not allow for causal inferences. In another longitudinal study [35], participants were compared to themselves. Attrition rate and practice effects might, however, have influenced the results.

As results of above-mentioned studies are both positive and negative, findings in this area are inconclusive.

### Cognitive development

In this review, studies focusing on the effects of music on children's cognitive abilities were subdivided into three categories, reflecting different aspects of cognitive development: (1) intelligence, (2) memory, and (3) attention and other executive function skills.

#### Intelligence

Several studies have explored the effects of music intervention on intelligence. Results from these studies suggest little or no beneficial effects. In an experimental design, Kaviani et al. (2014) [28] randomly allocated 60 5–6 year-old children to two groups, the experimental group receiving Orff music lessons and the other (matched for age-, sex-, and mother's educational level) receiving no lessons The authors demonstrated that after participating in the Orff music program for 3 months, children had significantly higher scores on the visual abstract reasoning, verbal reasoning and short term memory subscales of the Stanford—Binet Intelligence Scale compared to children, who did not receive any musical lessons. Schellenberg (2011) [24] and Bergman Nutley et al. (2014) [35] also reported positive associations between, respectively, music training and IQ and music training and non-verbal reasoning. In a longitudinal study, Moreno et al. (2011a) [25] used two subtests of the WPSI III (vocabulary and block design) to examine the influence of two interactive computerized training programs (music and visual arts) on, among other skills, verbal and spatial intelligence in 64 4–6 year-old children who were pseudo-randomly allocated to one of the two conditions. They found that children who participated in a computerized music training program showed enhanced performance on the measure of vocabulary knowledge. Not in line with above mentioned findings is the study of Mehr et al. (2013) [29]. They conducted an RCT to investigate the effects of parent-child music education on specific cognitive skills in preschool children. In experiment one, four-year-old children were randomly assigned to a music group (*n* = 15) or a visual arts group (*n* = 14). In experiment two, 23 children were randomly allocated to a music group and 22 children to a control group who did not receive music classes. Analyses with a combined music group (*n* = 38), the visual arts group and the control group revealed no significant effects on spatial-navigational reasoning, visual form analysis, numerical discrimination, and receptive vocabulary. Rickard et al. (2012) [42] failed to find an effect of increased classroom based music education on various cognitive measures. Bugos and Jacobs (2012) [26] evaluated the effects of a composition program, Composers in Public Schools (CiPS), on cognitive skills among 28 sixth-graders who were assigned to an experimental group (*n* = 15), receiving the CiPS program or a control group (*n* = 13), not participating in any musical courses. Results showed enhanced performance in arithmetic scores of the WISC-IV for the experimental group compared to the control group. No effect was found for vocabulary performance. Due to a relatively large variation in scores, enhancements for digit coding and symbol search subtests were not significant.

Only one out of the seven studies measuring the effects of music on intelligence, employed an RCT design including an active as well as a passive control group [29], which permits causal inferences to be made. No significant effects were found in that particular study. The two experimental studies reviewed [28] [42] yielded mixed results. While both used randomization [28] [sub experiment 2, 42], only one study, reporting no effect, included an active control group [42]. The remaining four studies, employed a quasi-experimental (longitudinal) design [24] [25] [26] or longitudinal developmental design [35], showed positive or partially positive effects. However, only one out of these four studies used pseudo-randomized group assignment, blinded outcome assessors and included an active control group. Schellenberg (2011) [24] and Bugos and Jacobs (2012) [26] both included a passive control group. Neither study matched participants on baseline variables. Despite the large sample size and duration of the study, caution is needed in interpreting findings of Bergman Nutley et al. (2014) [35], due to attrition and the possible influence of practice effects.

### Memory

A number of studies looked specifically at aspects of memory with mixed results. Degé et al. (2011) [31] demonstrated in a non-randomized, longitudinal design that after 2 years of extended music curriculum (ECM) training, short-term visual, and auditory memory scores for 16 9–11 year-old children, attending ECM training, had improved significantly, whereas no such increase was found in 25 children who did not attend ECM training. Roden et al. (2012) [33] conducted a quasi-experimental study where participants were allocated to a music program, a science program or a control group. Results showed that 25 children (mean age 7.73 years), who took part in a school-based music program, outperformed 25 children receiving extended natural science training and 23 children in a control group receiving no additional training, on verbal memory tasks. The authors failed to show a link between type of program and visual memory. Brodsky and Sulkin (2011) [1] reported positive effects of classroom handclapping intervention (HCST) on verbal memory. Results of a longitudinal study by Rickard et al. (2010) [30] showed significant enhancement of verbal learning and immediate verbal recall scores in 82 children (mean age 8.62 years) after ~1 year, but not 2 years after non-random allocation to an increased classroom-based instrumental music training, compared to 68 children (mean age 8.79 years), who did not receive training. In an experimental design, Martens et al. (2011) [32] focused on the effect of musical experience on verbal memory in 38 individuals with Williams syndrome, aged 6–59 years. Participants who had participated in formal music lessons scored significantly better on a verbal long-term memory task when the stimuli were sung than when they were spoken in comparison to those who did not have formal lessons, showing no benefit for either sung or spoken condition. Short-term memory did not appear to be affected by musical experience.

The five studies reviewed yielded mixed results. One experimental study [32] showed improved performance of participants who had participated in formal music lessons. However, generalizability of findings is low by including only participants with Williams syndrome, making the participants a non-representative sample. The remaining four studies, reporting positive or partially positive results, employed quasi-experimental (longitudinal) designs [1] [30] [31] [33]. However, in none of these four studies, participants were randomized or matched on potentially influencing variables, decreasing validity of findings. Blinded outcome assessors were used in one study [30]. Two out of the four studies included an active control group [1] or both an active and passive control group [33], allowing for a more detailed comparison. Although studies suggest potential benefits, the methodological limitations do not allow clear conclusions to be drawn about the effect of music and the part(s) of memory of which music can have an effect on.

#### Attention and other EF skills

The impact of music interventions on attention and several executive function skills was reported in seven studies with mixed evidence. One study of 102 7–12 year-olds Khalil et al. (2013) [37] found that, those, who were able to synchronize to a driving beat (in the context of a music class), were more attentive, showed less ADHD-like behaviors (rated by teachers) and performed better on an attention control task, in comparison to those who were less capable of synchronizing. Positive results have also been shown by Moreno et al. (2011a) [25], who reported enhanced performance on accuracy on a go/no-go task. Using a cross-sectional design, Zuk et al. (2014) [38] assessed (among other participants) 27 children (mean age 10 years) on a range of EF tasks. Fifteen instrumentally trained children, who started training on average at 5 years and had been studying their instruments on average 5.2 years, demonstrated heightened performance on coding, cognitive flexibility and processing speed tasks in comparison with 12 children without musical training outside the requirements of the general music curriculum in school. In contrast, Roden et al. (2014) [27], using a quasi-experimental design, investigated, among other skills, the effects of music lessons on processing speed abilities and visual attention in 7–8 year-old children over a period of 18 months. In the study, 345 children were assigned to the music training group (*n* = 192) or the natural science training group (*n* = 153). Children in the music group showed significant increases in information processing speed from T2 to T3. However, the level of significance was only associated with a small effect size. Although both groups improved their visual attention scores over time, these increases were stronger from T1 to T2 and T2 to T3 in children with natural science training as compared to children with music training. In a quasi-experimental study, Schellenberg (2011) [24] found that, with the exception of digit span, music training was independent of performance on phonological fluency, inhibition, problem solving, and planning and mental flexibility and rule switching. Bugos and Jacobs (2012) [26] found no effect of participation in a 4-month composition program on verbal fluency. Using an intervention design, Janus et al. (2016) [39] pseudo-randomly assigned 57 4–6 year-old children (matched on age and cognitive scores) to a 20-day music training (*n* = 28) or conversational French training program (*n* = 29) to compare the effects on executive control abilities. The one training-specific outcome found was that children in the French group showed broader improvement in visual search than children in the music program. For verbal fluency, grammatical judgement and visual search, all children performed significantly better after training.

Several studies suggested music training may improve various aspects of working memory. In one quasi-experimental, longitudinal study (Roden et al., 2014) [34], examined working memory performance in 25 7–10 year-old children who participated in a classroom-based, extended instrumental music training program and 25 children who participated in an extended science training program. Results showed significant gains in two out of three components of working memory performance in children who followed the music program for one-and-a-half-years in comparison to children who took part in the science training group. Positive associations between musical practice and working memory were also reported by Bergman Nutley et al. (2014) [35] and Zuk et al. (2014) [38]. Portowitz et al. (2014) [36] reported a significant enhancement in working memory scores in 62 9–10 year-old children after a 4-month participation in the (computerized) In Harmony program compared to 22 controls who did not participate in this program. The results of the study of Janus et al. (2016) [39] showed no effect of a music training program on spatial working memory.

The seven studies reviewed yielded mixed results of the influence of music interventions on attention and other EF skills. Positive correlations were shown by one study [37]. However, correlational studies do not allow for causal inferences. The remaining six studies were quasi-experimental (longitudinal) without randomization [24] [25] [26] [27] [38] [39]. Two of these six studies, reporting positive results, used pseudo random allocation of participants to groups [25] or matched participants on potentially confounding variables [38] but only one included an active control group [25]. Two other studies reported mixed and modest results, respectively [27] [24]. The sample size and the inclusion of an active control group can be considered as strength of one of them [27]. Of the remaining two studies [26] [39], reporting no evidence of beneficial effects of music, only one used blinded outcome assessors, pseudo randomization, and included an active control group [39]. Regarding working memory, there seems to be a hint of a positive influence of music based on the results of five studies [34] [35] [36] [38] [39]. However, studies were quasi-experimental (longitudinal) without randomization [34] [36] [38] [39] or longitudinal developmental [35]. Only three out of the five used pseudo random allocation [39] or matched participants on potentially confounding variables [36] [38]. An active control group was included by two out these five studies [39] [34], reporting no effects and mixed effects, respectively. The three other studies, all reporting positive findings, included a passive control group [36] [38] or compared the participants to themselves [35].

Although part of the evidence points to potential benefits, more research is needed to determine whether music can positively impact these skills.

### Academic performance

Studies exploring the effect of music on academic performance were subdivide into four categories: (1) school readiness, (2) classroom behavior and academic skills and (3) language, and (4) mathematics.

#### School readiness

One study focused, among other skills, on preschool children's school-readiness skills. The results of a quasi-experimental study of Ritblatt et al. (2013) [4] showed that participation in a music program had a positive effect on promoting a positive approach to learning. No effect was found for promoting academic skills.

Methodological limitations of this study are the lack over control over assignments of participants to conditions and the fact the sample may not be representative of the population as whole (i.c. higher SES and higher educational level), creating threats to validity. Information about the blinding of outcome assessors was not provided. The intervention was provided by trained teachers and parents, whose expectations may have influenced outcomes. Taking the limitations into account and the fact that the findings are based on one study only, accuracy and direction of the results should be interpreted with caution.

#### Classroom behavior and academic skills

There is no evidence that music can affect classroom behavior and academic skills. Pelham et al. (2011) [40] followed up 41 boys with ADHD and 26 normal comparison boys, who had never been referred for treatment of behavior problems (mean age 9 years) to examine the effects of music and video on classroom behavior and performance. Three distractor conditions (music, video, no-distractor) were randomly introduced for 24 days, varying on a daily basis (8 days in each distractor condition). Neither boys with ADHD or the control group were significantly distracted by music. Within the ADHD group, there were, however, considerable differences in response to the music such that some were adversely affected and others benefited relative to no-distractor. This study included males only, thereby eliminating a potential source of variability. Except for gender, participants were, however, not matched on any other variable. Outcome assessors were not blinded and the distractor conditions and no-distractor conditions may have been not much different. The accuracy and direction of the results should be interpreted with caution as findings are based on one study only.

#### Language

Several studies have explored the association between a music intervention or music training and performance on (specific) language skills respectively, with contradictory findings. With regard to first language skills, results of an RCT by Cogo-Moreira et al. (2013) [21] showed positive growing slopes in Portuguese language in the children who completed a 5-month music education program in comparison to the control children. Findings were in contrast to the results of Yang et al. (2014) [43] who, using a non-randomized, longitudinal design, examined the relation between long-term music training and, among other skills, academic development of Chinese language among 250 Chinese elementary school students (mean age 78 months). Children who took part in formal music training out of school around the beginning of semester three, were categorized as musician children (*n* = 77) whereas the remaining children, who had not received formal music training throughout this study, were categorized as non-musician children (*n* = 173). Music training was not related to the enhancement of performance on Chinese language.

Regarding second language abilities, Swaminathan and Gopinath (2013) [44] explored second-language abilities of musically trained children (*n* = 37)(mean age 100.55 months), who reported at least 3 months of music training and speaking a language other than English at home, and untrained children (*n* = 39)(mean age 98.89 months) and found that the musically trained children (mean length of training 17.63 months) performed significantly better on the tests of comprehension and vocabulary compared to their untrained counterparts. The advantage persisted even when the trained group only consisted of participants trained in Indian Classical music, indicating that the English L2 advantage was not merely because of an increased opportunity to learn new words from songs as Indian Classical music is written in Indian languages. Positive findings were also reported by Yang et al. (2014) [43], who found that musician children outperformed non-musician children on second language development.

Two studies [21] [43] reported contradictory results on the potential benefit of music on first language development. However, only findings from Cogo-Moreira et al. (2013) [21], conducting an RCT, allow for conclusions to be drawn about causality. Although the duration and sample size of Yang et al. (2014) [43] can be considered as strengths, participants were not randomized and a passive control was included. Another two studies reported positive results on second language development [43] [44]. Both studies made a comparison of the music group with a control group, who had no previous musical training. However, only one study [44] controlled for several baseline variables and used blinded outcome assessors, thereby increasing the validity of their findings.

#### Mathematics

Four studies have explored the effects of music on mathematics. A longitudinal study of Bergman Nutley et al. (2014) [35] yielded a positive association between music training (i.e., the number of hours per week of practice on instruments played) and performance on mathematics. Cogo-Moreira et al. (2013) [21] also observed positive growing slopes in math grades. In Yang et al's (2014) [43] study, however, music training was not related to performance on mathematics. Courey et al. (2012) [41] examined the efficacy of a music intervention aimed to teach fractions to third graders using a quasi-experimental design. Sixty-seven 8–11 year-olds were assigned by class to either a 6-week academic music intervention, administered during regularly scheduled mathematics instruction, or continued their regular mathematics instruction with their classroom teacher. The experimental group outperformed the comparison group on music notation knowledge and the mathematical fraction completion test (i.e., not previously introduced and improper fractions). No significant group differences were found on the mathematical fraction concept test.

The four studies reviewed yielded mixed results. One RCT [21] reported positive results. The remaining three studies were (longitudinal) quasi-experimental without randomization [41] [43] and longitudinal developmental [35]. Of these three studies, one found a positive association [35], one found partial positive results [41], and one found no relation [43]. Only one of these three studies included an active control group [41]. The duration and sample sizes of two out of these three studies can be considered as strengths [43] [35]. Although possible causal relations could be tested more easily, caution is needed in interpreting findings of Bergman Nutley et al. (2014) [35], due to attrition and possible practice effects.

### Other, non-musical, related skills

Two studies were identified that examined the effects of music on other, specific skills. Slater et al. (2015) [46] conducted a controlled, longitudinal study to investigate the effect of music training on speech in noise perception in 38 eight year-old children, randomly assigned to the music training program (*n* = 19) or the wait-list control group (*n* = 19). The authors reported a significant improvement of hearing in noise after 2 years of music training (Slater et al., 2015). Another longitudinal study of François et al. (2013) [45] tracked 24 eight year-old children after they were pseudo-randomly assigned to either a music training program or a painting program. They found that performance on both behavioral and electrophysiological measures of speech segmentation (i.e., the ability to extract meaningless words from a continuous flow of non-sense syllables) steadily increased across the testing sessions for the music group compared to the painting group.

Both studies, reporting positive results, employed a 2-year, longitudinal design and used valid (computer) measures to evaluate the performance of participants. Randomization, thereby reducing the risk of sampling bias, was used only in one study [46]. Information about the blinding of outcome assessors was not reported and only one out of the two studies employed an active control group [45]. Although sample sizes can be considered small, thereby limiting the external validity of findings, both studies propose an interesting direction for further research.

## Discussion

This review analyzed the evidence of 46 studies, dealing with five developmental domains, including the motor, social, cognitive, language, and academic domain.

With regard to the motor domain, the two studies identified suggested a positive influence of music interventions on specific motor skills (eye-hand motor sequences, discrete and continuous movements) [1] [2]. Due to the quasi-experimental design of the studies, the limited sample of participants and the inclusion of an active control group in one sub-experiment of one study only [1], clear conclusions cannot be drawn.

It cannot be concluded whether music interventions can positively influence social and emotional development as results of the nine studies reviewed [3] [4] [5] [6] [7] [8] [9] [42] are inconclusive. The findings of two experimental studies [3] [6] suggest a beneficial impact of music interventions on empathy and spontaneous cooperative and helpful behavior. The merging of the active and passive control group into one control group and the small sample size in one of the two studies, should, however, be taken into consideration. Positive findings of another study [7] turned out to be related to the level of cognitive functioning of participants in the music group. Caution is needed in drawing conclusions from the partially positive findings of Ritblatt et al. (2013) [4] and Schellenberg et al. (2015) [5] due to the design used [5] and the representativeness of the sample [4]. Two other experimental studies [8] [42] found modest effects and no effects, respectively, on social skills and self-esteem.

Regarding the language domain, 15 studies evaluating the impact of music interventions on phonological awareness and auditory processing and reading skills [12] [15] [16] [17] [18] [19] [20] [22] [23] [35], and no clear conclusions can be drawn in this area. The results of two randomized controlled trials are inconclusive. The results of four experimental studies with (stratified) randomization [13] [11] [20] [23] suggested beneficial effects of music interventions on reading skills [20] [23], however, not on phonological skills [11] [13]. Of these four studies, two included both an active and a passive control group [13] [23], allowing for a more comprehensive comparison. Of the remaining six studies, quasi-experimental (longitudinal) without randomization [10] [14] [16] [18] [22] and developmental longitudinal [35] in nature, three point to the beneficial effects of music [16] [18] [22]. The other three studies, including an active [10] or both an active and passive control group [14] or comparing participants to themselves [35], found no impact of music interventions.

With regard to the cognitive domain, seven studies reviewed provided insufficient information whether music can have a positive effect on intelligence. The results of an RCT [21] showed no effects and two experimental studies, only one of them including an active control group, yielded mixed results [30] [42]. Evidence of three quasi-experimental (longitudinal) studies [24] [25] [26] and longitudinal developmental study suggest a (partially) positive influence of music. However, an active control group was included in just one of these three studies [25]. Evidence of five (quasi-) experimental longitudinal studies seem to suggest potential benefits of music on memory. However, due to lowering generalizability of findings on one study [32], by including participants with Williams syndrome only, and methodological limitations of the other four studies (i.e., no randomization [1] [30] [31] [33] and/or no inclusion of an active control group [30] [31]), clear conclusions cannot be made. Among the six quasi-experimental studies exploring the potential influence of music on attention and EF skills, only two studies [25] [38] reported positive results. An additional five studies on working memory also seemed to suggest a positive influence. Whether or not an active control was included, the lack of randomization and the fact that working memory, attention and EF skills are difficult concepts to define, may have influenced the results obtained.

Regarding academic performance, research suggests some possible beneficial effects of music, although precise conclusions cannot be reached on the basis of reviewed studies. It cannot be concluded whether participation in a music program had a positive effect on promoting a positive approach due to the lack of randomization, the representativeness of the sample, the potential influence of parental and teacher expectations and the fact that the findings are based one study only [4]. The studies evaluating the impact of music interventions on first and second language development showed mixed findings. Regarding first language development, an RCT showed a positive effect, whereas a longitudinal study of Yang et al. (2014) reported no effects. Another two quasi-experimental studies showed improvement in second language performance [43] [44]. However, both studies included a passive control group. Of the four studies exploring the influence of music interventions on mathematics, one RCT reported positive effects [21]. Caution is needed in making causal inferences on the three remaining studies [41] [43] [35] due to the design used, the absence of randomization [41] [43] and, with regard to the study of Bergman Nutley et al. (2014) [35], attrition rate and possible practice effects. Evidence from the studies regarding the effectiveness of music on language and mathematics are reviewed separately. One can question whether there is a legitimate distinction between the two domains, as research suggest partial overlap between neural regions associated with language and arithmetic (Baldo and Dronkers, 2007; Cummine et al., 2014).

Five studies used a correlational design [9] [12] [15] [19] [37], reporting (partially) positive correlations between duration of music intervention and performance on reading and phonological awareness tasks, attention behavior, and self-esteem. Although correlational studies can provide an indication of a possible association between musical training and functioning domains, they do not allow for causal inferences.

The tool used for the methodological quality assessment allowed scoring between zero and five only. This makes a cut-off point difficult to determine. Although all domains included studies with lower quality scoring (two or less), these were more frequently found in the social and cognitive domain. This, however, does not mean that the results of these studies are invalid, but rather gives a direct for reading and interpreting them. The lower score of the study could often be explained by either unbalanced baseline characteristics, absence of randomization or missing information about blinding of outcome assessors or attrition rate. When analyzing the outcomes of the quality screening, one should take into consideration that it can be assessed with a broad range of tools. Upon applying the chosen tool, it was found that some of the items were difficult to relate to the studies at hand, but were more suitable for classical medical trials. Several criteria (including concealment of allocation and intention to treat) were negatively assessed in almost all studies, as they were not designed for the specifics of educational studies, where often it is impossible to ensure the rigid methodological quality: i.e., create double blind randomized trials.

When assessing the quality of the studied, there are several considerations regarding study design, music interventions, and the role of the teacher. In reporting on the participants, we found that little is mentioned about the intrinsic motivation of participants in the context of the intervention. As intrinsic motivation is associated with initiation and persistence of activities, level of effort and improved performance (Patall et al., 2008), gaining insight into the motivation of participants is important to be able to determine its impact on outcome measures. In some studies, interventions were partially provided by the authors themselves [1] [3] [6] [10] [22] [28] [36], or by parents and teachers [4]. As their expectations can have an effect on the performance of participants (the “Rosenthal effect”), one needs to be aware that observer bias rather than the intervention could cause the observed changes. Results of several studies might also have been affected by the “Hawthorne effect” i.e., a tendency of participants to alter their behavior because they are aware that they are studied. This effect cannot be ruled out or confirmed for diverse studies after screening.

Most of the study designs consisted of quasi-experimental and longitudinal designs and three studies were a RCTs. An RCT is considered as providing the strongest evidence of determining whether a cause-effect relationship exists between an intervention and outcomes (Sibbald and Roland, 1998) as assessment bias and confounding are minimalized. However, some research questions and settings don't permit random assignment of participants and questions may arise about the sample being representative enough of the population and the generalizability of findings to the field. As it is important to consider evidence from other methodologies as well to better understand the potential of music interventions in practice, only an RCT allows the observed effects to be causally attributed to differences between the intervention and the control group(s).

In reviewed studies, active and/or passive control groups were included in evaluating the effectiveness of a music intervention. Although showing whether participants benefit from an intervention compared to participants not receiving the intervention, passive control groups do not allow to test for intervention specific effects (Strobach and Karbach, 2016). Inclusion of an active control group, engaging participants in some training and activities during the intervention, can provide evidence as to whether an intervention is relatively more efficient than participating in another program (Karlsson and Bergmark, 2015), provided that the intervention and control group are matched on possible influencing factors and perform the same tasks.

Regarding the music interventions, studies were not uniform in their conceptualization of these music interventions. Some were very broadly defined and included listening, singing, instrumental playing, performing, movement, and musical creativity. While others, especially focused on the acquisition of non-musical skills, were more precisely defined and designed. Differences in musical content deserve attention in likely contributing to the outcomes of music interventions. Interventions in groups may have additional benefits of social interaction and motivation above the intervention itself compared to individual interventions which could have played a role in its final outcomes. In this review, the role of the teacher also emerged as a significant issue. 18 of the included studies employed (professional) music teachers and 16 reported at least partly positive outcomes. Teaching music requires many competencies. Strong teaching skills without musical skills and knowledge is not sufficient and vice versa. Research points not only to musical content knowledge, but also to pedagogical content knowledge and non-pedagogical professional knowledge (Ballantyne and Packer, 2004). By the way they teach, they play an important role in the teacher-child relationship which may have in turn implications for children's behavioral and academic adjustment (Furrer and Skinner, 2003). Therefore, teachers may also be an important factor in the context in which the effectiveness of music interventions is researched.

Another point of attention when describing the effects of music interventions on the development of children is the methodological accuracy and variety of different approaches the researchers took in their studies. Being the most powerful research design for evaluating interventions, further RCTs are needed to determine whether music interventions are effective in stimulating development in children. However, particularly in the domain of music interventions in schools, some requirements such as blinding, randomization, and controlling for potential sources of variability are often difficult if not impossible to achieve and RCTs may create an artificial situation in which findings may not always be applied to everyday practice. While we acknowledge the need for high-quality research methodology, it is important to find a balance between the externally imposed methodological standards and the drive to investigate a said phenomenon in its natural environment. As qualitative research can provide more insight into the characteristics of the intervention and can generate potential hypotheses for quantitative research, combining qualitative and quantitative research can give more comprehensive and integrated insights in potential effects of music interventions.

In conclusion, although the underlying mechanisms are not always clear, evidence of reviewed studies seems suggestive of some beneficial effects. Having a clearer view of effects and possible influencing factors may pave the way for further research on the influence of music on the developing child.

## Author contributions

ED the main author and executor of the research and participated in data collection and analysis as well as article writing process. ES was a second reviewer of the selected articles, contributed to development of methodology, data extraction, and analyses as well as final comments on the article writing. FF and SvH contributed to the idea of the development of the article as well as development of the research methodology and provided feedback during the article writing stage.

### Conflict of interest statement

The authors declare that the research was conducted in the absence of any commercial or financial relationships that could be construed as a potential conflict of interest.
